# The Oncolytic Effect of Respiratory Syncytial Virus (RSV) in Human Skin Cancer Cell Line, A431

**DOI:** 10.5812/ircmj.4722

**Published:** 2013-01-05

**Authors:** Vahid Salimi, Masoumeh Tavakoli-Yaraki, Mahmood Mahmoodi, Shahram Shahabi, Mohammad Javad Gharagozlou, Fazel Shokri, Talat Mokhtari-Azad

**Affiliations:** 1 Department of Virology, School of Public Health, Tehran University of Medical Sciences, Tehran, IR Iran; 2 Department of Clinical Biochemistry, School of Medical Sciences, Tarbiat Modares University, Tehran, IR Iran; 3 Department of Biostatic and Epidemiology, School of Public Health Tehran University of Medical Sciences, Tehran, IR Iran; 4 Department of Microbiology, Immunology and Genetics, Faculty of Medicine, Urmia University of Medical Sciences, Urmia, IR Iran; 5 Department of Clinical Sciences, Faculty of Veterinary Medicine, University of Tehran, Tehran, IR Iran; 6 Department of Immunology, School of Public Health, Tehran University of Medical Sciences, Tehran, IR Iran

**Keywords:** Oncolytic Viruses, Apoptosis, Skin Neoplasms, Flow Cytometry

## Abstract

**Background:**

Oncolytic viruses have become of noticeable interest as a novel biological approach for selectively infecting cancer cells and triggering apoptosis in a number of malignant cells. Many researches are devoted to characterize more viruses with oncolytic properties.

**Objectives:**

Evidences on the oncolytic feature of respiratory syncytial virus (RSV) are conflicting; therefore, this study was designed to elucidate the possible role of RSV on the modulation of cell growth and apoptosis in the skin cancer cells.

**Materials and Methods:**

Plaque assay was used to determine RSV titers. The cytotoxic effect of RSV in A431 (skin carcinoma cell line) was determined using MTT assay. The detection of apoptosis was performed via Annexin-V-FITC staining method and analyzed with flow cytometry.

**Results:**

The results indicated that A431 cell growth was inhibited following infection by RSV in a dose- and time-dependent manner. The most growth inhibitory effect of RSV was occurred at the MOI of 3, and 48 hour after infection. The inhibitory effect of RSV on the cell growth was accompanied by the induction of apoptosis in the skin cancer cells. The percentages of early and late apoptotic cells were increased following exposure to RSV in a concentration- and time-dependent manner.

**Conclusions:**

This study delineated the beneficial role of RSV for growth regulation of skin cancer cells and highlighted the involvement of RSV in the induction of apoptosis in A431 cells. These findings might conduct evidence into the oncolytic properties of RSV in the skin cancer. Further studies are required to indicate intracellular targets for RSV-induced apoptosis in skin cancer cells.

## 1. Background

Oncolytic viruses are able to infect and lyse cancer cells. They can also utilize the host cellular machinery to evade immune system, penetrate to the cell, and trigger cell death signaling cascades (1). The ability of oncolytic viruses manipulating same cellular pathways being disrupted during cancer development, brings out a novel pathway of research in cancer therapy (2).

Over recent years, oncolytic viruses have gained considerable interest as a promising molecular approach for apoptosis induction in cancer cells. Large body of investigations has been devoted to introduce more oncolytic viruses and related molecular mechanisms by which they affect progression of cancer cells (3-5). Wide spectrum of viruses has been proposed to regulate cell death so far, including herpes simplex virus (HSV) (6), measles virus (7, 8), Epstein-Barr virus (9), reovirus (10), Newcastle disease virus (NDV) (11), adenovirus (12), influenza virus (13), vesicular stomatitis virus (VSV) (14), coxsackie virus (15) and vaccinia virus (4).

Respiratory syncytial virus (RSV) belongs to the family of paramyxoviridae and is a negative single stranded RNA virus (16). RSV is the most potential lung pathogen causing lower respiratory tract infection during infancy. RSV infection is accompanied by various respiratory symptoms from mild common cold to bronchiolitis and pneumonia (17). RSV can lead to respiratory related mortality in adults with chronic lung inflammation or defected immune system (18). Over past decades, increasing evidence has delineated the oncolytic feature of RSV for apoptosis induction in cancer cells such as lung (19) and prostate (20). It has been shown that RSV can induce apoptosis in respiratory epithelial cells (21), and endoplasmic-reticulum-specific stress-activated caspase (caspase 12) is involved in RSV mediated apoptosis in A549 epithelial cells (19). It has been also demonstrated that RSV induced the selective disturbance of PC-3, human prostate cells, through down-regulation of NF-κB and stimulation of intrinsic apoptosis pathway (20). However, data on the effect of RSV on apoptosis in deferent types of cells is conflicting. Some studies suggest that apoptosis of neutrophils could be accelerated in the RSV bronchiolitis and provided explanation for the modulatory effect of RSV on neutrophil-induced damage of epithelial cells (22). Other studies, on the other hand, show that RSV can induce delay in neutrophil and eosinophil apoptosis via phosphatidylinositol 3-Kinase (PI3K) and NF-κB related pathways accompanied by up-regulation of anti-apoptotic markers (23). Therefore, implication of RSV in the signaling pathways related to apoptosis has been discussed controversially and further experiments are still required to delineate the oncolytic effect of RSV in different malignancies. It has been reported that skin neoplasm is the most diagnosed type of human cancer and rate of its mortality is increasing every year (24); therefore, finding efficient and novel therapeutic approaches is becoming an important concern for many research groups.

## 2. Objectives

In this regards, the biological significance of RSV in the molecular mechanisms leading to the cell death is important to be determined in skin cancer cells. We designed our current study to elucidate the possible effect of RSV on growth regulation and apoptosis in skin cancer cells. To achieve this, A431 was used as a human cell line of skin cancer. We examined the cytotoxic effect of RSV, the possible role of virus titer, and duration of virus infection on the growth inhibition. The relevance of RSV to apoptosis induction was determined, afterwards. Our results provided evidence that infection of cells by RSV resulted in a notable cell growth inhibition in A431 cancer cells.

## 3. Materials and Methods

### 3.1. Chemical Materials and Cell Culture

Dulbecco's modified Eagle medium (DMEM), phosphate-buffered saline (PBS), trypsin–Ethylenediaminetetraacetic acid (EDTA), penicillin, and streptomycin were obtained from Gibco (Rockville, USA). MTT [3-(4, 5-dimethyltiazol-2-yl)-2, 5-diphenyltetrazolium bromide], Annexin-V-FITC apoptosis detection kit, and dimethyl sulfoxide were purchased from Sigma-Aldrich (Munich, Germany). A431 (human skin cancer cell) was cultured in DMEM supplemented with 10% (v/v) heat inactivated fetal bovine serum (FBS), 100 U/ml of penicillin, and 100 µg/ml of streptomycin. Cells were incubated at a humidified atmosphere with 5% CO2 at 37 ºC. Cells collected at the confluence around 70-100% and exponential cells were employed freshly each time.

### 3.2. Virus Preparation and Titration

HEp-2 cells were used to prepare and grow RSV A2 strain (a gift from Louis Bont, Wilhelmina Children's Hospital, University Medical Center Utrecht, the Netherlands) and the virus titration was determined by plaque assay. To do that, a 10-fold serial dilution of RSV-A2 was prepared in DMEM to infect 80%–90% of the confluent monolayers of HEp-2 cells on 6-well plates for 1.5 h at 37 °C. Following virus adsorption, RSV infected cells were overlaid using 0.5% methylcellulose, which was contained DMEM medium supplemented with 2% fetal calf serum (FCS), and further incubated for 6 days at 37 °C. Then the methylcellulose overlay was aspirated and cells were fixed by 4% formaldehyde for 30 minutes at room temperature. Cells were stained with 0.2% crystal violet in 20% ethanol and light microscope was employed to count RSV plaques.

### 3.3. Cell Viability Assay: MTT Assay

MTT assay was used to evaluate the growth inhibitory effect of RSV. For determining the viability of treated and untreated cells, 15×103 cell/well of A431 were plated in 96-well plates in the presence of DMEM (supplemented with 10% fetal bovine serum , 100 U/ml of penicillin, and 100 µg/ml of streptomycin) in 5% CO2 at 37 ºC until nearly confluent. Cells were treated with the multiplicity of infection (MOI) of 1 and 3 of RSV for the indicated times (24, 36, and 48 hours). Afterwards, cells were exposed to 20 µl of MTT (5mg/ml in PBS) and incubated for 4 hours at 37 ºC. The formazan crystals were dissolved in 200 µl of dimethyl sulfoxide (DMSO), and the absorbance of plates were read by microplate reader at 570 nm (Bio-Rad, Hercules, CA, USA). The cytotoxic effect of each concentration of virus and the possible role of time-dependency were examined at least in three separate experiments. The growth inhibitory effect of RSV on the infected cells was compared to the control cells and was reported as a percentage of viable cells. 

### 3.4. Flow Cytometric Analysis of Apoptosis and Cell Death

Annexin-V and PI double staining methods were applied to quantitative apoptosis. The results were analyzed using flow cytometry according to the manufacturer’s specifications. Briefly, cells were treated with different MOI (1 and 3) of RSV and after indicated time, infected and non-infected cells were harvested at the density of 6×105 cells/ml. The collected cells were then washed twice by cold PBS, and the cell pallets were then re-suspended in 500 µl of 1X binding buffer. Re-suspended cells were undergone gentle vortex and stained with 5 µl of Annexin-V-FITC and 5µl of PI. Following short incubation for 10 min. in the dark at room temperature, the florescence of cells were analyzed by FACSCalibur flow cytometer (Becton Dickinson, San Jose, USA) using the in-built software ( BD Cell Quest software).

### 3.5. Statistical Analysis

The non-parametric one-way analysis of variance (ANOVA) with post hoc Dennet’s test was performed to compare and analysis of differences statistically using GraphPad Prism software. Data are expressed as mean ± SD of at least three separate experiments. A P value less than 0.05 (P < 0.05) was considered to be statistically significant for the differences and determined by asterisk in the corresponding figures.

## 4. Results

### 4.1. RSV Replication in Skin Cancer Cell Line A431

**Figure 1:**
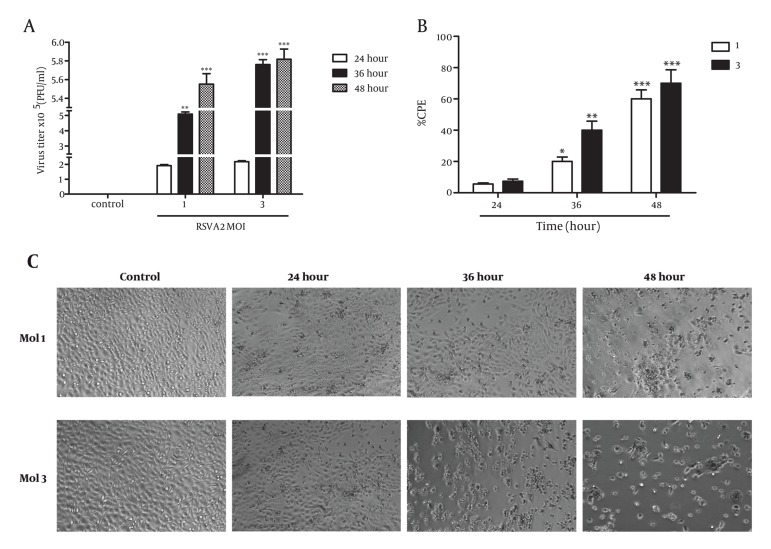
Propagation and Cytotoxicity of RSV in Human Skin Cancer Cell Line A431. (A) A431 cells were inoculated at different MOIs of 1 and 3 with RSV for 1.5 h, as indicated and cultured for 24, 36, and 48 h. At several time points post-infection, the virus titer in the culture medium was determined by plaque assay on HEp-2 cells. The results (Mean ± SD) represent at least 3 separate experiments. (B) The cytopathological changes of human skin cancer cell line A431 at different MOIs (1 and 3) and times (24, 36, and 48h) were determined by light microscopy (C). Percentage of cytopathic effect in A431 cell at various time points (24, 36, and 48 h) following RSV infection at MOIs of 1 and 3 were 6%, 20%, 61% and 8%, 41%, 72%, respectively. (Magnification: 4X)

There is a cell-specific difference in kinetics and efficiency of virus release related to the type of tumor cell lines (25). Therefore, we aimed first to check the ability of A431 human cell line to support the replication of RSV. Measurement of the virus release into the supernatant showed that A431 cells appeared to lag at the 24h point, but by 36h and 48h the titer of released virus was high at the indicated MOIs (Figures 1A and 1B). Percentage of cytopathic effect in A431 cells at the various time points (24, 36, and 48 h) following RSV infection at MOIs of 1 and 3 showed that A431 cells had low efficiency for RSV replication in a time-dependent manner (24h or less) (Figure 1C). The graph shows that virus is replicating increasingly in a time-dependent manner.

### 4.2. RSV Inhibited Cell Growth in the Skin Cancer Cell Line A431

**Figure 2:**
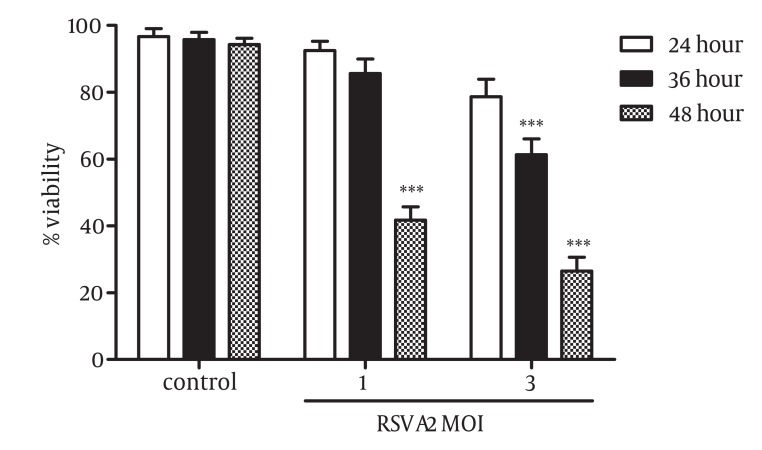
RSV Inhibited Cell Growth in Skin Cancer Cells, A431. Cells were infected with MOI of 1 and 3 of RSV for 24, 36, and 48 hours. The inhibitory effect of RSV on the growth of A431 cells were measured by MTT assay. RSV induced cell growth inhibition in a concentration- and time-dependent manner. The results (Mean ± SD) represent at least 3 separate experiments. Differences between RSV infected and non-infected cells were statistically analyzed by ANOVA (*** = P < 0.001)

The effect of RSV infection on growth and viability of skin cancer cells was evaluated. A431 cell line was treated with different MOIs of the virus (1 and 3) for 24, 36, and 48 hours and the viability of infected and control cells were measured using MTT assay. As shown in Figure 2, RSV infection resulted in a considerable decrease in the percentage of A431 viable cells. The results indicated that RSV inhibited the viability of A431 cells in a dose- and time-dependent manner. The growth inhibitory effect of RSV at MOI of 1 was 58.25% after 48 hour (P = 0.0001) and no significant inhibitory effect was observed for this concentration of the virus after 24 and 36 hours. At the MOI of 3, RSV reduced the viability of cells to about 32% (P=0.0005) and 73.5% (P=0.0001) after 36 and 48 hours, respectively. 

### 4.3. RSV Induced Apoptosis in A431 Skin Cancer Cells

**Figure 3:**
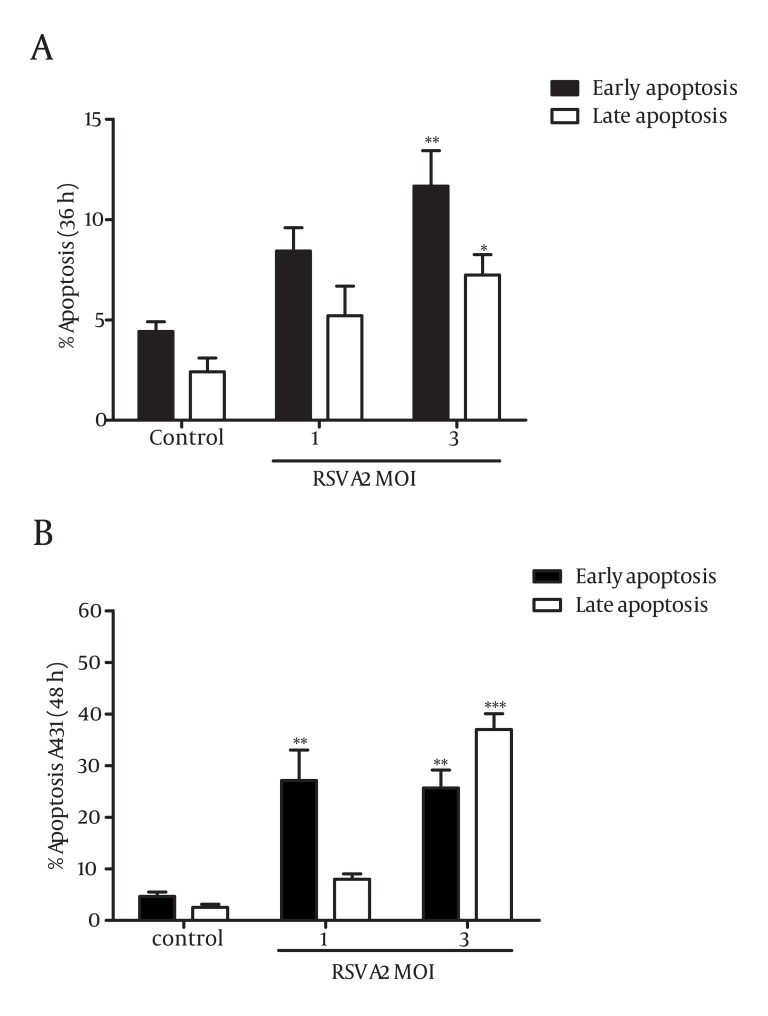
The Effect of RSV on the Induction of Apoptosis in Cancer Cell Line, A431. Cells were infected with different concentrations of RSV (MOI of 1 and 3) for 36 (A) and 48 (B) hours. Annexin-V and PI staining methods were employed for detection of apoptosis using flow cytometry. The percentages of early and late apoptotic cells were increased following exposure to RSV in a concentration- and time-dependent manner. The results (Mean ± SD) represent at least 3 separate experiments. Differences between RSV infected and non-infected cells were statistically analyzed by ANOVA (* = P < 0.05, **= P < 0.01, ***= P < 0.001)

To ascertain whether or not the inhibitory effect of RSV on A431 cells was associated with the induction of apoptosis, Annexin-V and PI double staining were performed and analyzed using flow cytometry. According to the method, early apoptotic cells are characterized as Annexin-V positive, and PI-negative cells, and late apoptotic cells are recognized as Annexin-V positive and PI-positive cells. Subsequently, controls are considered as Annexin-negative and PI- negative cells. A431 cells were infected with MOI of 1 and 3 of RSV for 24, 36, and 48 hours and analyzed by flow cytometry. The results indicated that an increase in the rate of early (P = 0.0071) and late (P = 0.033) apoptotic cells were detected in A431 cells following infection by MOI of 3 of RSV in 36 hours (Figure 3.A). Cells infected by MOI of 1 of the virus revealed a significant increase of early apoptotic cells after 48 hours (Figure 3.B). A remarkable elevation in the percentage of early and late apoptotic cells were also observed at the MOI of 3 of virus after 48 hours (P = 0.0037, P = 0.0001) (Figure 3.B). Our data demonstrated that infection of A431 cells by increasing concentrations of RSV was accompanied by a notable shift of the viable cells to the apoptotic cells in a time- and dose-dependent manner. The percentage of A431 apoptotic cells (early and late) was determined from 13.64% to 18.89% after 36 hours and from 35.08% to 66.62% after 48 hours in the indicated MOI of 1 and 3, respectively. It should be noted that no significant differences were observed on the percentage of apoptotic cells between infected and non-infected cells after 24 hours (data not shown).

## 5. Discussion

A considerable promotion in the knowledge of oncolytic viruses and their emergence to trigger apoptosis cascades has led many research groups to characterize the possible roles of such viruses in different types of malignancies (5, 26). The balance between cell proliferation and cell death is under a precise regulation and is necessary to be maintained for the homeostasis of normal cells (26). Oncolytic viruses have been contributed to induce apoptosis in cancer cells and emerged as novel and promising anti-cancer agents (4). Multiple lines of evidence underlined the ability of RNA and DNA viruses (4, 6, 8-15, 20) to suppress cancer cell growth and induce apoptosis. It was well established that RSV induced the apoptosis in lung and prostate cancer cells (18, 27); however, it will be important to delineate the biological relevance of RSV on triggering death-signaling pathway in other cancer cells.

The heavy burden of skin cancer-related mortality, lack of effective treatment, and better-tolerated anti-cancer approaches (28) motivated us to elucidate the possible role of RSV in growth regulation of skin cancer cells. We designed our experiments to characterize the effect of RSV on the regulation of cell growth and induction of apoptosis in cancer cell line of the skin. A431 was taken as a skin cancer cell and provided us more evidence on the biological role of RSV in different tissues. Treatment of A431 with increasing MOI of RSV resulted in a notable decrease in the percentage of viable cells. The growth inhibitory effect of RSV was occurred in a concentration- and time-dependent manner in cancer cell line of human skin, A431. Our results revealed that elevation in the viral load was associated with the remarkable reduction in the population of viable cells (Figure 2). It can be concluded that replication of virus is a beneficial agent for elimination of cancer cells. This is in agreement with the positive effect of RSV on the growth inhibition of androgen dependent (27) and independent (20) prostate cancer cells. It was shown that the rate of RSV replication was higher in tumorigenic compared to non-tumorigenic prostate cancer cells, and the increased RSV burden was accompanied by the growth inhibition of more prostatic cancer cells (27). The same results were reported on the pro-apoptotic role of RSV on respiratory epithelial cells (19). To further determining whether cytotoxic effect of RSV on A431 cell line was related to the induction of apoptosis, Annexin-V/PI double staining method was performed using flow cytometry. A significant increase in the number of apoptotic cells were observed following infection of cells by RSV (Figure 3). A marked enhancement in the number of apoptotic cells after treatment by RSV was closely related to the virus load that were in line with the effect of RSV on the induction of apoptosis in neutrophils (22), prostate (20, 27), and lung cancer cells (19, 21).

It has been elucidated that RSV fusion protein F was responsible for the oncolytic effect of RSV in lung. Fusion protein F triggered caspase cascade and caused p53 phosphorylation in respiratory epithelial cells (29). Apparently, molecules involved in virus replication might have crucial effect on destroying tumor cells, and it would likely be important to delineate their precise roles.

The result of our study revealed that RSV was involved in the cell growth inhibition and apoptosis induction in the skin cancer cells, which was associated with the virus load and time (Figures 3A and 3B). However, data on the oncolytic effect of RSV in literature has been discussed controversially. It has been reported that RSV stimulated phosphatidylinositol 3-kinase, increased NF-κB activity (23) at the early steps of infection in airway epithelial cells, and gave rise the cell survival. Interestingly, simultaneous treatment of cells by PI3-K inhibitor and infection by RSV increased the cytotoxic effect of RSV and caused cell death as early as 12 hour after infection (23). Additionally, it has been shown that RSV inhibited apoptosis of granulocyte through activation of PI3-K, NF-κB, and p38 via interaction with intercellular TLRs (30).

The conflicting data on the pro- versus anti-apoptotic role of RSV appear to be explained by tissue specificity of RSV and multiple possible molecular approaches that might be contributed to the significant role of RSV in tumor cells. Further studies are required to expand our knowledge on the biological effect of RSV on the tumor cells and get more insight into the signaling pathways underneath RSV infection. In conclusion, we demonstrated that RSV mediated the growth inhibition in cancer cell line of skin. The observed inhibition of cell growth was associated with the induction of apoptosis, and was notably under the control of virus replication. This study provided additional evidence that RSV imposed a potent oncolytic effect on cancer cells, highlighting the relevance of RSV on the cell death pathways in skin malignancies. However, further investigations are required to elucidate the molecular mechanism of RSV-mediated apoptosis.
